# Maintenance of certification in Internal Medicine: participation rates and patient outcomes

**DOI:** 10.3402/jchimp.v2i4.19753

**Published:** 2013-01-07

**Authors:** Dolores Buscemi, Helen Wang, Michael Phy, Kenneth Nugent

**Affiliations:** Department of Internal Medicine, Texas Tech University HSC, Lubbock, TX, USA

**Keywords:** certification, recertification, internal medicine, patient outcomes, mortality

## Abstract

The clinical practice of internal medicine continues to evolve with the addition of new information and new technology. Most internists in practice will have erosion of their knowledge after they complete training unless life-long learning occurs. The American Board of Internal Medicine (ABIM) began to issue time-limited certification in 1990 and asserts that the Maintenance of Certification (MOC) program promotes the professional development of internists. However, the available medical literature does not provide strong support for the assumption that internists with certification or recertification have better patient outcomes. This relationship between recertification and patient outcomes needs more study. In addition, the participation in the Maintenance of Certification program by internists with lifetime certifications has been low, and recertification by leaders in internal medicine has also been relatively low. Some physicians in practice have concerns about the relevance of the program and the cost. Our review suggests that the ABIM needs to review its current Maintenance of Certification program and make changes to enhance its clinical relevance and educational value. We suggest that professional development should be based on focused reviews of the current literature, which is immediately relevant to clinical practice, and that recertification could be based on completion of modules and more frequent, less onerous testing.

In 1990, the American Board of Internal Medicine (ABIM) began to issue time-limited certifications. This decision reflected the ABIM's assertion that its diplomates involved in such a program would maintain competency in clinical medicine and up-to-date medical knowledge. The ABIM outlined these goals for recertification in 1991 as follows: (1) Improve the quality of patient care; (2) set standards of clinical competence for the practice of internal medicine; and (3) foster the continuing scholarship required for professional excellence over a life-time of practice (1). Intuitively these goals seem reasonable. The question we ask is whether there are actual data to support this decision and its goals. Does the medical literature demonstrate a relationship between certification status or recertification status and patient outcomes? (2). We also reviewed the participation rates of internists with time-limited and lifetime certificates in the Maintenance of Certification (MOC) program to determine the value placed on recertification by current diplomates.

## Methods

We carried out PubMed searches using the MeSH terms *Internal Medicine*, *Patient Outcomes*, *Hospital Mortality*, and *Certification*. We also used these terms as text words and used recertification as a text word. The search strategy combined *Internal Medicine* AND *Certification* (or *recertification*) AND *Hospital Mortality* (or *Patient Outcomes*) using MeSH terms when available; these searches were then repeated using same strings as text words. We reviewed the reference lists from articles that reported information on certification (or recertification) and patient outcomes, and we used the PubMed related articles algorithms associated with pertinent articles. We also searched the bibliography of ES Holmboe who has published multiple articles in this area and is an employee of the ABIM. We verified certification of individual physicians using the ABIM website (www.abim.org). We identified editors and officers in various internal medicine organizations using official websites: ABIM (www.abim.org), Annals of Internal Medicine (www.annals.org), American College of Physicians (www.acponline.org), and ACGME-RRC Internal Medicine (www.acgme.org).

## Discussion

### Patient outcomes and certification status

Chen et al. studied the association between the care of acute myocardial infarction (AMI) (aspirin, beta-blocker administration, and 30 day mortality) and the board certification status of the treating physicians (Table 1) (3–7). They reviewed the charts of 101,251 Medicare patients cared for by family practitioners, general internists, and cardiologists. Board-certified physicians were more likely to meet the quality of care indicators than those who were not board certified but did not have better mortality rates in their patients. Norcini et al. also studied mortality in patients with AMI (4). They compared board-certified family practitioners, general internists, and cardiologists with non-certified physicians and found that certified physicians had better outcomes in patient mortality. Kelly and Hellinger retrospectively reviewed the influence of selected characteristics on the survival of in-hospital cardiac patients (5). These patients were divided into three categories: patients who underwent coronary artery bypass graft (CABG), patients who underwent cardiac catheterization without CABG, and patients with diagnosis of AMI who did not have surgery. The board certification status did not influence patient outcomes for the first two patient groups but did influence the outcomes in the third group. Board-certified internists had 3.1% fewer patients with an AMI die than non-certified physicians, and board-certified family practitioners had 4.2% fewer patients with an AMI die. In 1989, Ramsey et al. studied the predictive value of ABIM board certification on several competencies (6). They prospectively evaluated 185 certified and 74 non-certified internists who had completed training within the last 5–10 years. The physicians were given a written examination of 119 questions developed by the ABIM. The other components of evaluation included a self-administered patient questionnaire, an evaluation by their professional associations in the form of a questionnaire, and a review of their medical records. The board-certified physicians had higher exam scores and higher ratings of their clinical skills by professional societies. Based on the medical record review, board-certified internists did a better job with preventive counseling, but the difference was small. There was no difference in the patient survey questionnaires. Pham et al. studied how well primary care physicians delivered preventive services (7). The board-certified physicians performed better on delivering these services (i.e., colon cancer screening, mammogram), but these differences were small. In addition, overall performance was poor (e.g., approximately 50% of women had a mammogram during the year). Prystowsky has studied the effects of certification and experience in patient outcomes with colon surgery (8, 9). His studies demonstrate that both certification and experience affect primary outcomes, including in-patient mortality, complications, and length of stay. These studies on surgical performance have clear advantages when compared to studies with internists since the outcomes are more easily defined and quantifiable, and they suggest that practice-related experience has important effects on patient outcomes. This experience might not be easily identified with standardized tests. All these studies in the literature depended on retrospective chart reviews and, therefore, provide information about associations. They are unavoidably limited by confounding factors and missing information.

**Table 1:** Studies with Outcomes/Board Certification

Authors	Data source	Specialties studied	Quality measures	Results
Chen et al. 2006	Data from CCP and AMA Physician Master profile 101,251 Medicare patients	Internal medicine Cardiology Family medicine	Patients admitted with AMI: Admission ASA Discharge ASA Admission B-blocker Discharge B-blocker	Board-certified internists and cardiologists performed better than non-boarded; no difference in mortality
Kelly and Hellinger 1989	National patient abstract data for 1977	Board-certified IM/FM and non-board-certified	Mortality associated with acute MI	Board-certified internists had 3% fewer deaths in hospital
Norcini et al. 2002	Patients with AMI 40,684 hospital admissions	Board-certified and non-board-certified internists, family practitioners, cardiologist	Mortality after acute MI	15% reduction in mortality with board certified
Ramsey et al. 1989	Internists who had completed training in the last 5–10 years	185 board certified/74 non-board-certified internists	Management of specific diseases Preventive counseling Ratings by Professional Society Patient questionnaire Written exam	Certified internists had significantly higher exam scores. Rating of clinical skills by Prof Soc were significantly higher. No difference in patient satisfaction No difference in chronic disease management Modest difference in preventive counseling favoring certified
Pham et al. 2005	24,581 Medicare beneficiaries; claims data from 2001	3660 general internists and family practitioners	Delivery of preventive services to Medicare patients ≥ 65	Board-certified physicians did better statistically at ordering HgbA1C, colon cancer screen, mammograms

We found two studies that examined patient outcomes and maintenance of certification status (10–12). In 2008, Holmboe studied physicians who had completed the MOC requirement and divided them into four quartiles based on their examination scores (10). They examined standard quality of care indicators for diabetes (HgbA1C, lipids, eye exam), mammogram rates for women aged 65–74, and timely lipid testing in patients with known coronary disease and found a correlation between quartile and performance on these measures. Patients who were cared for by a physician in the top quartile were 17% more likely to receive all three diabetes QOC measures; women were 14% more likely to be referred for screening mammogram. The lipid testing did not show a difference, possibly explained by concomitant care by cardiologists. However, in this study physicians in the top quartile did not meet performance goals adequately. Physicians who scored over 600 on the examination met all three of the diabetes quality measures only 40% of the time. Hess et al. studied the relationship between cognitive skills based on MOC examination scores and quality of care scores in diabetes care using information from the ABIM Diabetes Practice Improvement Module (PIM) (12). There was a significant association between the MOC score and the diabetes composite score index, but the overall model only explained 13% of the variability in the diabetes care score. In addition, information in the PIM may not reflect actual clinical practice since this information from PIM is collected during ABIM recertification. This may motivate some participants to *get the right answer*. Overall, this study suggests that multiple factors influence patient care and that test scores do not predict complex practice behaviors, and that it provides information on only one diagnosis.

Insurance companies, hospitals, and governmental agencies also influence physician performance and patient outcome. Chen et al. reported that ASA use in AMI on admission was approximately 50% during 1994–1996 (Table 1) (3). Williams et al. examined 18 quality of care measures for AMI, heart failure, and pneumonia using quarterly data from US hospitals for the period 2002–2004 (13). In July 2002, the Joint Commission on Accreditation of Healthcare Organizations implemented standardized performance measures. By the third quarter of 2002, the performance was significantly better. For example, patients admitted with AMI who received ASA on admission went from 78% in the first quarter of the study to 93% in the eighth quarter of the study. These changes occurred in the hospitals with the lowest performance at the baseline survey. Therefore, hospitals and the Joint Commission have been instrumental in improving these measures. The studies on the effect of board certification on performance measures in Table 1 do not report any effect with a magnitude similar to this change. Does the maintenance of certification program have any additional effect on core measure performance?.

The available studies do not demonstrate that physicians with board certification provide substantially better care. This is likely explained by the effect of external influences on patient care and by the fact that physicians completing residency training may have similar overall performance regardless of any particular standardized test result. Has recertification been a successful endeavor? Based on two studies, physicians who do better on the MOC examination meet quality of care standards better, but the overall effect is modest. Can we conclude that physicians who have not participated in MOC would not perform as well on these measures? There are no published studies comparing physicians who have completed the MOC program and those who have not.

### Participation in the MOC program

According to the ABIM's official website, 79% of physicians with time-limited certificates (1990–1997) have participated in the MOC program. The ABIM does not report data on physicians who have lifetime certificates. We approached the question of whether or not internists with lifetime certificates were participating in the MOC with two samples. We developed a sample of community physicians from Lubbock, TX, because we could identify all general internists in our home city. This city has a population of 260,000 with 55 general internists. Thirty-six have time-limited certificates, and seven have lifetime certification. None of the physicians with lifetime certificates have participated in the MOC program. We also calculated the recertification rate of the internal medicine leadership in various organizations using information collected from various websites in July 2009, which was approximately 20 years after the change in certification to a time-limited process. The initial ABIM task force on recertification has a recertification rate of 18% (3/17). The ABIM Board has a recertification rate of 20% (6/20). The editorial board for the *Annals of Internal Medicine* has a recertification rate of 9% (2/22). The ACP Governors have a recertification rate of 4% (2/54). The ACP Board of Regents has a recertification rate of 8% (2/26). The ACGME-RRC Internal Medicine Committee has a recertification rate of 0% (0/12). These are the statistics for internal medicine recertification only; the rates for recertification in subspecialty areas are slightly higher (Table 2). We repeated part of this analysis in June 2012. Twenty-six members of the ABIM board had lifetime certification, and six (23%) have voluntarily recertified. Thus, the participation by senior leadership in internal medicine has been unusually low; this seems surprising since many of these individuals promoted the initial process. *The New England Journal of Medicine* recently completed a survey which asked readers to advise a physician with a lifetime certificate in internal medicine and endocrinology as to whether or not this individual should enroll in the ABIM Maintenance of Certification Program (14, 15). A total of 2,512 votes were cast; 63% of the respondents did not recommend enrollment in this current program. Reasons for this decision included cost, which appeared to outweigh the educational benefit, and the lack of relevance to day-to-day patient care. These readers argued for refinement of the MOC process to make it more topical and pertinent to practicing physicians.

**Table 2:** Percentage of members with non-time-limited certificates who have recertified

Organization	Certified general medicine *N*	Recertified general medicine *N* (%)	Certified subspecialty *N*	Recertified subspecialty *N* (%)
ABIM	30	6 (20)	17	13 (76)
Task force	17	3 (18)	11	3 (27)
Annals editorial	22	2 (9)	5	1 (20)
ACP regents	26	2 (8)	7	0 (0)
ACP governors	54	2 (4)	18	0 (0)
ACGME	12	0 (0)	8	0 (0)
Total	161	15 (9%)	66	17 (25%)

## Conclusions

The field of internal medicine constantly acquires new studies and information. Ramsey and co-authors reported an inverse correlation between performance on an ABIM type test and the number of years out of residency training (16). To limit this erosion in medical knowledge and to help reassure the public about the quality of health care, the ABIM began to issue time-limited certification in 1990. The program has evolved into the MOC and has the potential to help clinicians gain and retain medical knowledge. Levinson and Holmboe recently reviewed the evolution and current status of the Maintenance of Certification program (17). They quote the same articles we reviewed in Table 1 to demonstrate the relationship between certification status and patient outcome. They report that the ABIM Practice Improvement Modules are analyzed by unique statistical software. Although this approach is probably a practical necessity, it cannot provide the same analysis as an experienced expert clinician could and the real benefit of this activity is not as clear as they claim. They note that approximately 75% of diplomates start the recertification process in the ninth year of the 10-year cycle. Hence, MOC hardly represents continuous professional improvement. Finally, they admit that less than one percent of diplomates with lifetime certificates participate. The ABIM had previously stated that ‘it is convinced that as recertification becomes the norm, older internists will choose to voluntarily recertify’ (18). This has not happened to date and, based on the participation rates of internal medicine leaders and the sentiments in the NEJM poll, is not going to happen. This lack of participation should raise fundamental questions about the process. Finally, the president of the Alliance for Academic Internal Medicine has claimed that the public and payers do not consider the 10-year cycle to be a credible method to evaluate competency and that the MOC must evolve into a continuous process (19). The basis for this conclusion is unclear, but it does represent additional concerns about the MOC process.

In summary, the relevance and importance of the ABIM MOC program is a difficult calculation and will likely vary from individual to individual and from clinical practice to clinical practice. The ABIM is not the only organization with a strong interest in physician performance, and other organizations, such as state licensure boards, could develop competing programs to ‘certify’ current competency. Chaudhry outlined the expected changes in the maintenance of a licensure process which will be required by the Federation of State medical Boards (20). This process will have requirements like the ABIM recertification process but will likely be easier. Also, hospitals and/or insurance companies could develop other educational and professional development activities that are more directly relevant to patient care and local services. The ABIM should be concerned about the possibility that diplomates with time-limited certificates will opt out of the MOC program, especially if more requirements are placed on physicians to practice medicine. Therefore, the ABIM should undertake additional studies to correlate patient outcomes with MOC status. These studies should compare physicians who choose to recertify with those who do not and should evaluate the effect of other professional development activities on patient outcome. These studies are obviously complicated and will likely require more than small-scale academic studies. At a minimum they should include a prospective design with prespecified outcomes and analysis. The critical characteristics of certified physicians which drive outcomes will be hard to determine but could be modeled using propensity analysis. These studies will have difficulty capturing the contributions of day-to-day clinical experience in patient outcomes.

We offer the following recommendations to promote professional development:

1. The ABIM should develop a directed reading program to focus on recent advances in internal medicine. For example, a committee could select one new article every two weeks with a clinical scenario highlighting the new information. This would provide a focused update and could form the basis for an annual test that would cover recent medical literature. These articles could be specialty specific.
2. The ABIM should administer an annual test in place of the current 10-year secure test. This test would have fewer questions and would focus on recent advances in internal medicine. Since diplomates cite cost as a reason they are reluctant to recertify, this new test would be paid for annually, and this would reduce costs to a more modest annual fee. This could be built into ACP dues and would provide the CME needed for state licensure.
3. The ABIM should develop diaries that help internists critique their daily clinical activities. This would have no cost and requires only time which would be relevant to current patient care. This activity could replace the ABIM modules.
4. The ABIM should directly observe physician performance in the clinic or hospital every 5–10 years. This would allow direct feedback and might identify areas needing remediation. We understand this could be practically difficult and costly, but the information obtained would be worth the investment. In addition, this information would provide the basis for educational programs sponsored by the ABIM or ACP. Alternatively, the ABIM could require internists to attend and pass clinical skills workshops for updates. The Advance Cardiac Life Support program sponsored by the American Heart Association is one possible model for clinical skill review. This activity updates physicians and improves performance; it also saves lives.

We think these changes will enhance the MOC program by lowering costs, by encouraging practice review by individual physicians and experts, and by continuously focusing on the current medical literature. These changes will make the MOC process more meaningful and useful for physicians with both time-limited and lifetime certificates and will increase participation. This approach will help maintain the wealth of existing knowledge of internists. However, demonstration of clinical skills remains a difficult transaction, and the development of the best process to support this activity largely depends on the commitment of internists to their profession.
